# Meta-Analysis of the Efficacy of Laparoscopic Pyeloplasty for Ureteropelvic Junction Obstruction via Retroperitoneal and Transperitoneal Approaches

**DOI:** 10.3389/fped.2021.707266

**Published:** 2021-07-29

**Authors:** Fengming Ji, Li Chen, Chengchuang Wu, Jinrong Li, Yu Hang, Bing Yan

**Affiliations:** Kunming Children's Hospital, Kunming, China

**Keywords:** ureteropelvic junction obstruction, laparoscopic, pyeloplasty, retroperitoneal, transperitoneal

## Abstract

**Objective:** This study aimed to evaluate the clinical efficacy of laparoscopic pyeloplasty (LP) for ureteropelvic junction obstruction (UPJO) via retroperitoneal and transperitoneal approaches.

**Method:** A systematic literature search on keywords was undertaken using PubMed, Cochrane Library, Embase, China Nation Knowledge (CNKI), and Wanfang. The eligible literature was screened according to inclusion and exclusion criteria. Meta-analysis was performed by using RevMan 5.0 software.

**Results:** According to the inclusion and exclusion criteria, 12 studies were identified with a total of 777 patients. Four hundred eight patients were treated with retroperitoneal laparoscopic pyeloplasty (RLP), and 368 patients were treated with transperitoneal laparoscopic pyeloplasty (TLP). The meta-analysis results showed that the two approaches were similar in terms of presence of postoperative hospital stay, postoperative complication, the rate of conversion, and recurrence (*p* > 0.05). The operative time in the TLP group was significantly shorter than the RLP group (MD = 16.6; 95% CI, 3.40–29.80; *p* = 0.01). The duration of drainage was significantly shorter (MD = −1.06; 95% CI, −1.92 to −0.19; *p* = 0.02), and the score of postoperative visual analog score (VAS) was significantly lower in the RLP group than in the TLP group (MD = −0.52; 95% CI, −0.96 to −0.08; *p* = 0.02).

**Conclusion:** Both approaches have good success rates and low postoperative complication rates. RLP provides a shorter duration of drainage and lower VAS score, but it takes more operative time than TLP.

## Background

With the popularity of prenatal ultrasound, the rate of diagnosis of hydronephrosis has increased in fetal and prenatal. There are many causes of hydronephrosis such as ureteropelvic junction obstruction (UPJO), vesicoureteral reflux (VUR), or ureterovesical junction obstruction. Ureteropelvic junction obstruction (UPJO) is the most common cause of congenital hydronephrosis (1). The standard surgical technique is dismembered pyeloplasty (Anderson–Hynes procedure) for UPJO, which was first performed successfully by Anderson and Hynes in 1949. It has the obvious advantages for long stenosis segment, presence of stones, and crossing vessels (2). With the continuous development of modern minimally invasive technology, laparoscopic pyeloplasy (LP) has become a more beneficial choice for the patients with UPJO than open surgery because of the advantages of excellent visualization, minimal trauma, rapid postoperative recovery, good cosmetic result, and a nearly successful rate compared with open pyeloplasy (3, 4). LP can be performed though retroperitoneal and transperitoneal approaches. To compare the advantages and disadvantages of the two approaches, this study consulted relevant literature and performed a meta-analysis.

## Methods

### Search Strategy

We searched PubMed, Embase, CNKI, and Wanfang. Studies were restricted to English and Chinese language published before January 1, 2020. The following search terms were used using the Boolean operator terms “AND” and “OR”: “laparoscopic pyeloplasty,” “laparoscopic disconnected pyeloplasty,” “Ureteropelvic junction obstruction,” “UPJO,” “retroperitoneal,” and “transperitoneal.”

### Inclusion Criteria and Exclusion Criteria

#### Inclusion Criteria

(1) Interventions: laparoscopic ureteroplasty was performed through retroperitoneal and transperitoneal approaches. (2) Intervention subjects: unilateral UPJO patients. (3) Outcomes: postoperative time, hospital stay, postoperative complication, duration of drainage, visual analog score (VAS), the rate of conversion, and recurrence. (4) Study types—randomized controlled studies or retrospective studies. (5) For the studies published by the same unit, the latest one would be included.

#### Exclusion Criteria

(1) Approaches involved only retroperitoneal or transperitoneal. (2) The intervention subjects included patients with bilateral UPJO. (3) Outcome cannot be obtained. (4) Full text is unavailable. (5) The treatment includes robotic-assisted surgery and open surgery. (6) Literature with a quality evaluation result of <7 or low quality according to the Newcastle–Ottawa Scale (NOS) quality evaluation scale (5) and the Cochrane Collaboration's tool (6).

### Study Selection and Quality Evaluation

In selecting studies for inclusion, a review of all relevant article titles and abstracts were conducted before an examination of the full published texts. Two professional reviewers reviewed the articles for eligibility and quality and extracted the data independently. Data were collected on standard collection tables. Extracted data included author's name, nation, published year, study type, patients' characteristics, and relevant outcomes. Disagreement was resolved by consensus with the intervention of a third reviewer.

For the quality assessment, the Newcastle–Ottawa Scale (NOS) quality evaluation scale and the Cochrane Collaboration's tool were used for non-randomized controlled trials and randomized controlled studies, respectively.

### Statistical Analysis

All meta-analyses were carried out in RevMan 5.0 software, and *p* < 0.05 meant the difference was statistically significant. The continuous variables were described by standardized mean difference (SMD) and 95% confidence interval (95% CI), and the dichotomous variables were described by odds ratio (OR) and 95% CI. Evaluated by *Q*-test, heterogeneity was considered if *p* > 0.1 or *I* < 50%, and the random effect model was adopted. If *p* < 0.1 or *I* > 50%, it indicates that there was heterogeneity, and the fixed effect model was adopted. For the continuous variables, if only the median and value range were provided in the included studies, the mean and standard deviation were calculated according to the formula of Hozo (7).

## Result

### Study Characteristics

**Figure 1:**
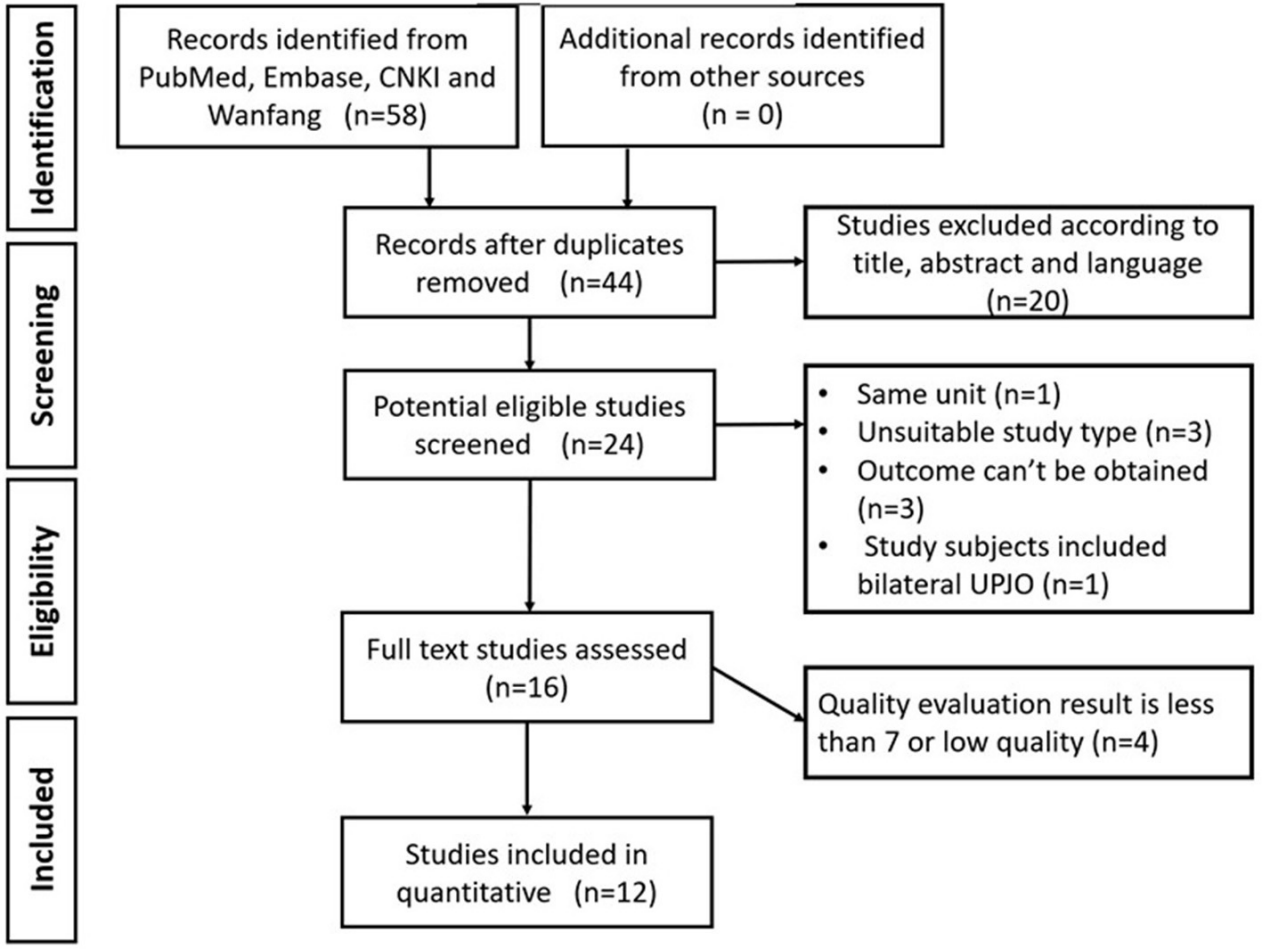
Search process of the studies.

A total of 44 studies were retrieved. According to the inclusion and exclusion criteria, there were 12 studies that were included in the present study, of which 7 studies were in English, and 5 studies were in Chinese. A total of 777 patients were involved among the 12 studies, 408 patients were treated with RLP, and 369 patients were treated with TLP (The basic characteristics of the included studies are shown in Table 1, and the search process of the studies is shown in Figure 1).

**Table 1:** The basic characteristics of the included literature.

**References**	**Nation**	**Year**	**Study type**	**RLP/TLP**				
				**Side: eft/right**	**Sex: male/female**	**Age:**	**Mean follow-up period**	**Quality evaluation**
Abunaz et al. (8)	France	1999/10–2008/10	RS	14:17/16:18	12:19/15:19	36.94 ± 17.92/37.11 ± 16.75	48.9	8
Badawy et al. (9)	Egypt	2010/06–2012/09	RCT	/	11:8/14:5	/	10	High
Hemal et al. (10)	India	1999/10–2002/03	RS	4:8/7:5	8:4/9:3	26.3 ± 10.46/22.9 ± 9.87	11	9
Liu (11)	China	2012/09–2017/09	RS	20:8/21:9	17:11/18:12	27.12 ± 4.56/28.43 ± 3.25	/	9
Qadri and Khan (12)	India	2000/01–2009/08	RS	16:19/5:7	25:10/8:4	27.3 ± 11/32 ± 10.18	22/48	9
Shen et al. (13)	China	2012/04–2017/03	RCT	26:17/23:20	29:14/31:12	38.18 ± 3.05/39.11 ± 3.01	/	High
Shoma et al. (14)	Egypt	2002/02–2006/01	RCT	14:6/11:9	10:10/11:9	34 ± 15/29 ± 13	20/23	High
Singh et al. (15)	India	2008/01–2012/12	RCT	31:25/30:26	32:24/30:26	24.93 ± 3.94/24.79 ± 3.96	31	High
Xu and Li (16)	China	2013/01–2015/01	RCT	/	27:13/26:14	26.45 ± 4.45/26.34 ± 4.35	20	High
Zhai et al. (17)	China	2011/06–2015/05	NRCT	31:25/27:15	34:22/28:14	30.8 ± 12.8/27.2 ± 11.9	26/24	9
Zhang (18)	China	2010/01–2012/12	RS	22:18/24:16	22:18/28:12	22.41 ± 5.18/26.67 ± 4.59	18	7
Zhu et al. (19)	China	2009–2011	RS	16:12/13:9	16:12/9:13	30.6 ± 13.5/37.5 ± 8.25	11/10	8

### Meta-Analysis Results

#### Operative Time

**Figure 2:**
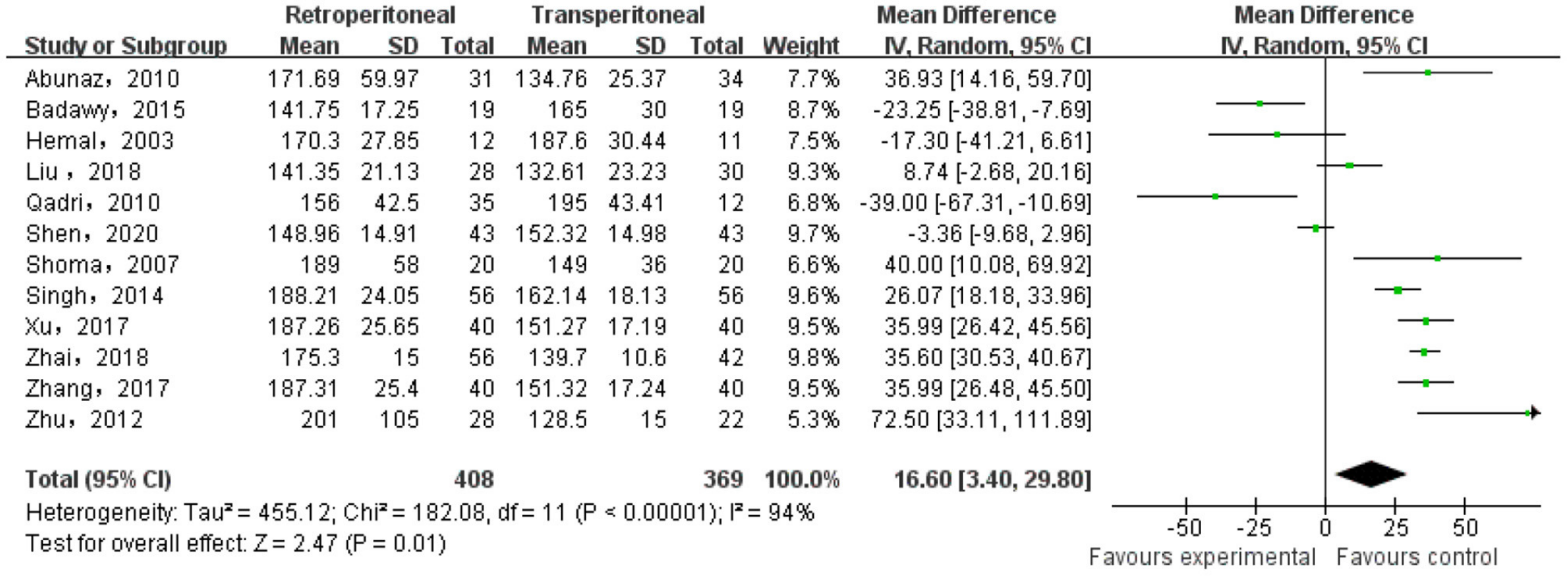
Meta-analysis of the operative time.

There were 12 studies that reported the operative time of the two groups. The heterogeneity test result was *p* < 0.0001, *I* = 94%, and the random effect model was adopted. The meta-analysis result showed that there was significant difference in the operative time between the two groups (MD = 16.60; 95% CI, 3.40–29.80; *p* = 0.01) (Figure 2).

#### Postoperative Hospital Stay

There were 12 studies that reported the postoperative hospital stay of the two groups. The heterogeneity test result was *p* < 0.0001, *I* = 91%, and the random effect model was adopted. The meta-analysis result showed that there was no significant difference in hospital stay between the two groups (MD = −0.21; 95% CI, −0.54–0.12; *p* = 0.21).

#### Duration of Drainage

**Figure 3:**
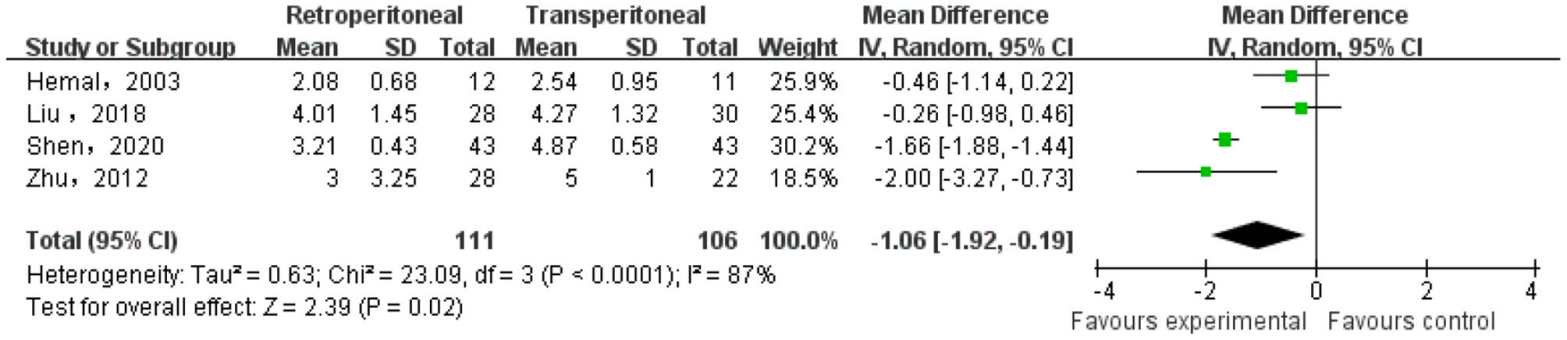
Meta-analysis of the duration of drainage.

There were four studies reported the duration of drainage of the two groups. The heterogeneity test result was *p* < 0.0001, *I* = 87%, and the random effect model was adopted. The meta-analysis result showed that there was significant difference in the duration of drainage between the two groups (MD = −1.06; 95% CI, −1.92 to −0.19; *p* = 0.02) (Figure 3).

#### Visual Analog Score

**Figure 4:**
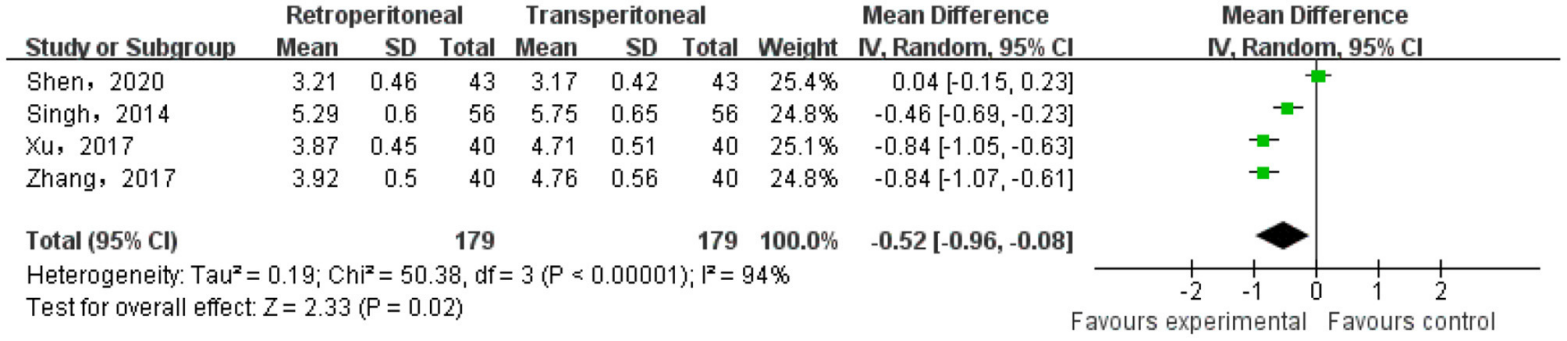
Meta-analysis of the visual analog score (VAS).

There were four studies that reported the VAS of the two groups. The heterogeneity test result was *p* < 0.0001, *I* = 94%, and the random effect model was adopted. The meta-analysis result showed that there was a significant difference in the VAS between the two groups (MD = −0.52; 95% CI, −0.96 to −0.08; *p* = 0.02) (Figure 4).

#### Postoperative Complication

There were seven studies that reported the postoperative complication of the two groups. The heterogeneity test result was *p* = 0.51, *I* = 0%, and the fixed effect model was adopted. The meta-analysis result showed that there was no significant difference in the postoperative complication between the two groups (OR = 1.19; 95% CI, 0.62–2.28; *p* = 0.60).

#### Conversion Rate

There were six studies that reported the conversion rate of the two groups. The heterogeneity test result was *p* = 0.36, *I* = 7%, and the fixed effect model was adopted. The meta-analysis result showed that there was no significant difference in the conversion rate between the two groups (OR = 1.86; 95% CI, 0.67–5.16; *p* = 0.23).

#### Recurrence

There were six studies that reported the recurrence of the two groups. The heterogeneity test result was *p* = 0.99, *I* = 0%, and the fixed effect model was adopted. The meta-analysis result showed that there was no significant difference in the recurrence between the two groups (OR = 1.23; 95% CI, 0.55–2.74; *p* = 0.62).

#### Publication Bias

**Figure 5:**
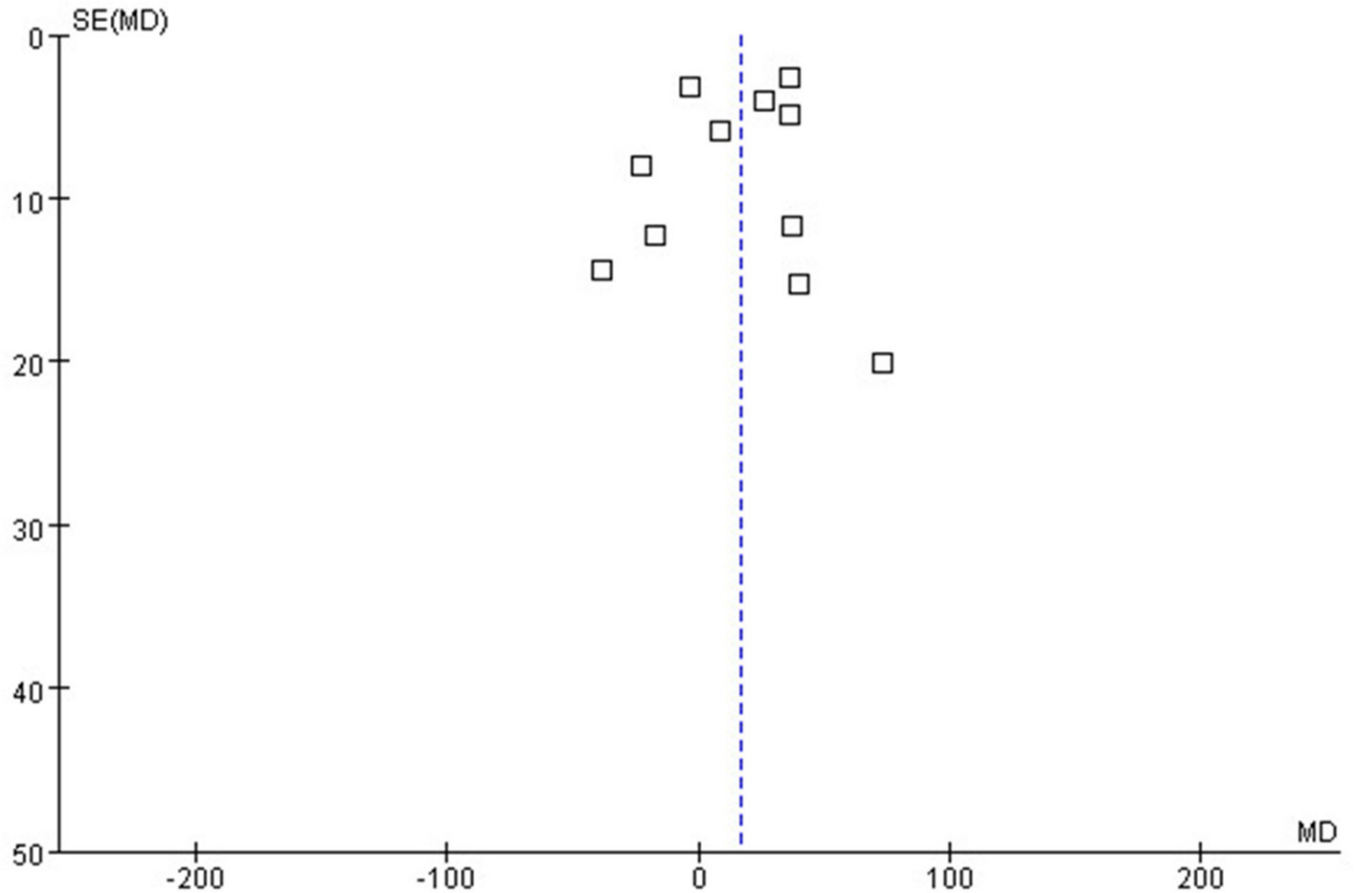
The Funnel plot.

In the bias analysis, the effect index SMD was used as the abscissa axis and SE (SMD) as the vertical axis to draw an inverted funnel plot (see Figure 5). The results showed that the funnel plot was not completely symmetrical on the left and right, suggesting that there might be publication bias in the included literatures in this study.

## Discussion

UPJO is a common disease in pediatric urology with an incidence of about 1/2,000 in newborns, and the ratio of the men to women is 2~3:1 (20). UPJO usually reduces the free flow of urine from the renal pelvis to the ureter, causing dilation of the renal pelvis and calyces and hydronephrosis (21). Ureteropelvic junction stenosis, crossing vessel, and ureteropelvic junction valve and stone are also important causes of UPJO. Ureteropelvic junction stenosis is the most important cause of congenital UPJO in newborns, which can impair renal function and eventually lead to renal parenchymal atrophy (22). Lack of smooth muscle, collagen deposition, increased connective tissue, and decrease in the proportion of interstitial cells of the Cajal are the pathological characteristics of ureteropelvic junction stenosis. According to the study of Bady et al. (23), the stenosis segment is related to the increase in acetylcholinesterase activity and norepinephrine response.

Surgical intervention for UPJO is aimed at removing of obstruction segment, relieving of pain, and preserving of renal function (4, 24). There are many indices that have been used to identify the need for surgery, such as SFU grade 3 or 4, continued expansion of the renal pelvis collection system, a renal cortex <5 mm, a single kidney with decrease in GFR, and symptom of pain (25, 26). Regrettably, there has been no reliable criterion that could be used in risk stratification and decision making with UPJO. Most researches support that the reduction in cortical thickness and increased severity of hydronephrosis are important signs of fibrosis of renal parenchyma and reduced glomerular numbers (27); however, in the study of Huang et al., (28) it was pointed out that the degree of hydronephrosis did not significantly correlate with the number of affected glomeruli. Mercapto-acetyl-triglycine and dimercaptosuccinic acid can objectively reflect the degree of kidney damage, but they usually need sedation and repeated evaluation in infants or younger children. Pavlaki et al. (29) proved that the level of urinary NGAL and serum cystatin C are remarkably decreased from the preoperative to the postoperative period, and they could be reliable biomarkers to distinguish the kidney condition among patients with severe and mild hydronephrosis.

There are many methods for treating UPJO, including endopyeloplasty, endopyelotomy, and pyeloplasty, but pyeloplasty is the most reliable, which is currently recognized as the gold standard for the treatment of UPJO (30). Up to now, the overall success rate of the open pyeloplasty is over 90%, and the recurrence of postoperative hydronephrosis usually occurs within 2 years after the operation. Chow et al. (24) pointed out that preoperative renal function <30%, history of endopyelotomy, and early urinary leakage were the risk factors for surgical failure.

According to the results of the meta-analysis, there were no significant difference between the two approaches on postoperative hospital stay, complications, conversion rate, and recurrence. RLP took more operative time than TLP, and the difference was statistically significant (MD = 16.60; 95% CI, 3.40–29.80; *p* = 0.01). Since the transperitoneal approach requires to cut the mesentery through the medial or lateral colon to enter the retroperitoneal cavity, it takes more time to expose the pelvis. Wu et al. (31) believed that the retroperitoneal approach will be more conducive to shortening the operative time with the familiarity of the surgeon with the anatomy of the retroperitoneal cavity. According to the results of the present study, RLP can significantly shorten the time of postoperative drainage and reduce the score of postoperative VAS (MD = −1.06; 95% CI, −1.92 to −0.19; *p* = 0.02; MD = −0.52; 95% CI, −0.96 to −0.08; *p* = 0.02), which may be related to the shorter route of retroperitoneal approach, with less interference to abdominal organs, faster recovery of gastrointestinal function, and low incidence of intestinal obstruction.

Open pyeloplasty has been widely accepted as the prior choice for UPJO, with a success rate of >90% in most reports (32). Since the LP in adults and children were first successfully performed in 1993 and 1995, respectively, it has gradually replaced open pyeloplasty as the preferred option for UPJO (33, 34). Most researchers support that LP is beneficial and advantageous to old patients, but for infants younger than 6 months, opinions are different (35, 36).

Nowadays, the application of laparoscopy in pediatric urology has been developed for 30 years. Laparoscopy seems to be an established technique for children. LP may be applied with transperitoneal and retroperitoneal approach. TLP can provide a larger space for free movement of instruments and intraoperative suturing. Meanwhile, the anatomical marks are easier to identify for surgeons. However, due to the stimulation of urine to the intestinal and the disturbed abdominal cavity, the rate of bowel-related complications, including abdominal organ injury and postoperative intestinal obstruction, is increased (1). Which surgical method is better is still controversial; some scholars argued that if the renal pelvis dilated more than 6 cm, with large or multiple renal stones, pelvic kidney, or horseshoe kidney, TLP was easier and safer than RLP (37). Because the infants have a high sensitivity at CO_2_ effects, theoretically, increased intra-abdominal pressure and hypothermia, TLP seems to be safer for infants to decrease the intra-abdominal pressure and hypothermia through shortening of the operative time (36, 38). Unfortunately, postoperative hypercapnia was not reported in the literature included in the study.

In terms of TLP, the surgical approach remains controversial too. TLP included paracolic sulci approach and mesentery approach, and the option of surgical procedure usually depends on the location of the lesion. Due to the right renal pelvis and ureter, which are often located at the right colic flexure, UPJO on the right is recommended with the optimal paracolic sulci approach. During the operation, only the peritoneum of the lateral side of the right colon is cut, and the right colon is pushed medially to expose the renal pelvis and ureter. Due to the left colic flexure position, which is higher, covering the kidney, and the mesenteric just covering the left UPJ, the left mesentery approach is not only helpful in identifying the renal pelvis but also can avoid excessive dissection and dissociation of the left descending colon and perirenal fascia, shorten the operation time, relieve surgical trauma, relieve postoperative pain, and accelerate postoperative recovery (39, 40).

There were some limitations to this study that should be noted. On the one hand, not all of the studies included were RCT; it caused an inevitable selection bias in the study. On the other hand, there was limited documentation of follow-up; of the 10 studies assessed, 2 studies gave no length of follow-up and 3 studies published on a follow-up of <12 months. It affected the outcome of the long-term postoperative complications.

## Conclusion

RLP and TLP have the same results in postoperative complications, conversion rate, and recurrence, but RLP has potential benefit to make the patients recover faster after the operation as it can reduce the time of postoperative drainage and postoperative VAS. It is hard to say which approach is better because RLP takes more operative time and needs a longer learning curve, so the surgeon should choose the appropriate operation according to personal preference and experience during the early practice. For experienced surgeons, RLP seems to be a more beneficial choice for patients.

## Data Availability Statement

The original contributions presented in the study are included in the article/supplementary material, further inquiries can be directed to the corresponding author/s.

## Author Contributions

FJ collected and analyzed data and drafted the original manuscript. LC collected data and participated in to amend the manuscript. CW collected and analyzed data. JL collected data. YH analyzed data. BY designed present study and amended the manuscript. All authors contributed to the article and approved the submitted version.

## Conflict of Interest

The authors declare that the research was conducted in the absence of any commercial or financial relationships that could be construed as a potential conflict of interest.

## Publisher's Note

All claims expressed in this article are solely those of the authors and do not necessarily represent those of their affiliated organizations, or those of the publisher, the editors and the reviewers. Any product that may be evaluated in this article, or claim that may be made by its manufacturer, is not guaranteed or endorsed by the publisher.
